# Low Molecular Weight Heparin (LMWH) Improves Peritoneal Function and Inhibits Peritoneal Fibrosis Possibly through Suppression of HIF-1α, VEGF and TGF-β1

**DOI:** 10.1371/journal.pone.0118481

**Published:** 2015-02-27

**Authors:** Juan Li, Zhi Yong Guo, Xian Hua Gao, Qi Bian, Meng Jia, Xue Li Lai, Tie Yun Wang, Xiao Lu Bian, Hai Yan Wang

**Affiliations:** 1 Department of Nephrology, Changhai Hospital, Second Military Medical University, Shanghai 200433, China; 2 Department of Colorectal Surgery, Changhai Hospital, Second Military Medical University, Shanghai 200433, China; University of Utah, UNITED STATES

## Abstract

**Background:**

Peritoneal fibrosis is the major cause of ultrafiltration failure, and intraperitoneal administration of Low Molecular Weight Heparin (LMWH) was reported to protect peritoneal function. But the exact mechanism of its influence on peritoneal structure and function is still unknown.

**Methods:**

A fibrosis model of rat was established by intraperitoneal (IP) administration of PD fluid and Erythromycin Lactobionate. Fifty-two rats were randomly divided into 6 groups: (1) normal control group (CON, n = 6); (2) normal saline group (NS, n = 10); (3) high-glucose group (GLU, n = 10); (4) heparin group (HEP, n = 6); (5) low dose LMWH group (LLMWH, n = 10); (6) high dose LMWH group (HLMWH, n = 10). Two hour peritoneal equilibration test was performed after 28 days of intervention. The peritoneum, mesentery and omentum were harvested, and evaluated by Hematoxylin-Eosin and Masson Trichrome staining. The expressions of HIF-1α, VEGF and TGF-β1 in parietal peritoneum were detected by IHC and RT-PCR (Reverse Transcriptase Polymerase Chain Reaction).

**Results:**

Compared with group CON and NS, ultrafiltration volume and D2/D0 glucose in group GLU decreased significantly, D/Purea (Dialysate-Plasma ratio of urea), D/Palb (Dialysate-Plasma ratio of albumin), peritoneal thickness, neoangiogenesis and inflammatory reaction increased significantly (all P<0.05). Administration of heparin and LMWH markedly alleviated these above pathological changes. The protein and mRNA levels of HIF-1α, VEGF and TGF-β1 increased significantly in group GLU, and decreased significantly after administration of LMWH in a dose-dependent manner.

**Conclusions:**

LMWH ameliorates peritoneal function and inhibits peritoneal fibrosis, possibly through suppression of HIF-1α, VEGF and TGF-β1.

## Introduction

Continuous Ambulatory Peritoneal Dialysis (CAPD) is a standard therapy for patients with end-stage renal failure. However, long-term CAPD would induce chronic sterile inflammatory state, loss of mesothelial cells, submesothelial thickening, neoangiogenesis and peritoneal fibrosis, and cause functional deterioration of the peritoneal membrane, with increasing peritoneal permeability to small solutes and reduced ultrafiltration volume (UF) [1]. The non-physiological environment of Peritoneal Dialysis Fluid (PDF), such as hypertonic glucose, glucose degradation products, lactate, acid pH and peritonitis[2,3], have been identified as responsible for the functional deterioration and peritoneal fibrosis.

Peritoneal fibrosis is the major cause of ultrafiltration failure and death for CAPD patients [4]. Chronic hypoxia is the major cause of peritoneal fibrosis [5]. Hypoxia inducible factor -1 alpha (HIF-1α) is a specific transcription factor in hypoxia condition. Having more than 60 direct target genes, HIF-1 α plays an important role in angiogenesis, extracellular matrix metabolism, inflammatory reaction and peritoneal fibrosis [6]. Heparin is a highly sulfated polysaccharide synthesized by mast cells. Besides its anticoagulant effect, heparin has a number of immunomodulatory and anti-inflammatory activities, including modulating various growth factors and cytokines [1]. Low molecular weight heparin (LMWH), which is superior to heparin in terms of improved bioavailability, prolonged half-life, improved efficacy and minimal interference with normal blood coagulation function, is widely applied in hemodialysis patients [1]. Sjoland[7] reported that LMWH significantly reduced peritoneal permeability to small solutes and elevated UF. Sjoland[8] also demonstrated that long-term intraperitoneal (IP) administration of LMWH retarded local and systemic inflammation. A recent study indicated that LMWH alleviated inflammation and fibrosis in rats’ lung tissue with chronic obstructive pulmonary disease (COPD), through inhibiting the expression of HIF-1α and its downstream target genes VEGF (Vascular Endothelial Growth Factor) [9], which is a well-known growth factor that promotes neoangiogenesis. TGF-β1 (Transforming Growth Factor—beta 1), which was recognized as a potent fibrosis factor, is also reported to be involved in peritoneal fibrosis and ultrafiltration failure [2]. Our previous clinical trials had confirmed that LMWH could protect peritoneal structure and function, and that HIF-1α was up-regulated in CAPD patients depending on the extent of peritoneal fibrosis.

On the basis of the above evidences, we hypothesize that LMWH may ameliorate peritoneal function and inhibit peritoneal fibrosis through suppression of HIF-1α, VEGF and TGF-β1. Therefore, we designed this animal experiment, using a peritoneal fibrosis rat model induced by high glucose, to observe the effects of LMWH on peritoneal structure and function, and to explore possible mechanism.

## Materials and Methods

This study was approved by the institutional review board of Changhai Hospital, Second Military Medical University, Shanghai, China. The study complies with current ethical consideration.

### Drugs and Major Reagents

LMWH (0.4ml:4100AXaIU, France GSK Corp.), 4.25% peritoneal dialysis fluid (Baxter Corp.), HIF-1α rabbit polyclonal antibody (Abcam Corp.), VEGF rabbit polyclonal antibody (Abcam Corp.), TGF-β1 rabbit polyclonal antibody (Abcam Corp.) and EnVision Immunohistochemical Kit (Dako, Denmark).

### Animal Model

All rats were male and weighted 180–220 grams. Rats had free access to standard diet and water. Rats were kept in a pathogen-free laboratory, and experiments were performed following guidelines of Second Military Medical University and China. After 7 days of adaptive feeding, 52 male SD rats (body weight, 180–220g), provided by the experimental animal center of Second Military Medical University, were randomly assigned to six groups. These groups included a normal control group (CON, no intervention, n = 6); a normal saline group (NS, daily IP injection of 20 ml of 0.9% normal saline, n = 10); a high-glucose group (GLU, the model group, daily IP injection of 20 ml of 4.25% PDF, n = 10); a heparin group (HEP, additional daily IP injection of heparin 1mg on the basis of the model group, n = 6); a low-dose LMWH group (LLMWH, additional daily IP injection of LMWH 62.5IU on the basis of the model group, n = 10), and a high-dose LMWH group (HLMWH, additional daily IP injection of LMWH 125IU on the basis of the model group, n = 10). The injection site was sterilized with an iodophor before each IP injection. Except for group CON, all rats in the other five groups received daily peritoneal dialysis (PD) for 28 days, and reveived IP injection of Erythromycin Lactobionate 62500IU at d7 (the seventh day of intervention), d14, d21 and d28 to accelerate peritoneal fibrosis and prevent peritonitis.

### Sample Collection and Testing

After cessation of peritoneal dialysis for 48 hours, 2 hours peritoneal equilibrium test (PET) were performed in all rats. Each rat received IP injection of 25ml of 4.25% PDF, and 2 hours later rats were anesthetized by intramuscular injection 10% chloral hydrate (0.3ml/100g). And then the abdominal cavity were opened along the linea alba. After laparotomy, 2ml of ascites were obtained with disposable sterile syringe under sterile conditions for biochemistry test. Remained intra-abdominal fluid was dried by gauze, and the gauze was weighed. The ultrafiltration volume (UF) was (the weight of wet gauze—the weight of dry gauze) + drainage volume—25. The blood was collected through the inferior vena cava. The serum and dialysate were preserved. The glucose concentration of the dialysate was determined and used to calculate the D2/D0 (the ratio of 2 hours to 0 hour glucose concentration of the dialysate in PET). The mesentery, greater omentum and parietal peritoneum far from the injection site was gathered. And then the inferior vena cava was exposed, and blood was retrieved from the inferior vena cava rapidly with a syringe. The rat died rapidly ahead of the termination of anesthetics effect. This euthanasia method make sure that rat die rapidly and painlessly.The retrieved blood were preserved for further study. Hematoxylin and Eosin (HE) and Masson Trichrome Staining were performed to observe the pathological changes. The expression levels of HIF-1α, VEGF and TGF-β1 in the parietal peritoneum were evaluated by immunohistochemistry (IHC) and RT-PCR.

### Pathology of the parietal peritoneum and greater omentum

The paraffin-embedded peritoneum specimens were dried at 60°C to dissolve the wax after it was sliced. HE and Masson Trichrome Staining were performed to observe pathological changes. After HE staining, ten horizons (×100) were randomly selected in each slice to calculate the mean vascular unit in greater omentum, and ten horizons (×400) were randomly selected in each slice to calculate the mean of inflammatory cells and peritoneal thickness in parietal peritoneum. After Masson staining, ten horizons (×400) were randomly selected in each slice to calculate the mean of peritoneal thickness.

### IHC of HIF-1α, VEGF and TGF-β1 in parietal peritoneum

IHC was performed using Envision IHC technique (Dako Kit, Denmark). After deparaffinization and rehydration, antigen retrieval was done with citrate buffer (0.01 mol/L, pH = 6.0) by Water bath (98°C 25min). The tissue sections were pre-incubated with 0.3% hydrogen peroxide and 20% normal goat serum to block nonspecific reactions. A primary antibody (HIF-1α 1:200, VEGF 1:100 and TGF-β1 1:200) was incubated on the slides for 2 hours in a humidified chamber. HRP-conjugated secondary antibody was applied neat for 30 min. The sections were stained with DAB and counterstained with Mayer’s hematoxylin, mounted with Pertex mounting medium, and coverslipped. Ten horizons (×400) were randomly selected in each slice and photographed. Appearance of brown granules in the target cell is regarded as positive reaction. Each image was scored as negative (0 score), weak positive (1 score), positive (2 scores), strong positive (3 scores). The average score of the ten images was regarded as the staining score of the slide.

### Reverse Transcriptase Polymerase Chain Reaction (RT-PCR)

Total RNAs were extracted from fresh frozed tissue using Trizol reagent (Invitrogen, USA) and reverse transcribed using ReverTra Ace qPCR RT Kit (TOYOBO, Japan), according to the manufacturers’ instructions. The resulting complementary DNA (cDNA) was used for real-time PCR using the SYBR Premix Ex Taq II (Applied TaKaRa, Japan) in triplicates. PCR and data collection were performed on the TP800 qPCR System (Takara, Japan). All data were normalized to an endogenous control (GAPDH). The relative value for the target gene compared to its calibrator is expressed as 2^-(Ct-Cc)^ (Ct and Cc are the mean threshold cycle differences after normalizing to GAPDH)). Primers used in PCR are showed in Table 1.

**Table 1:** Primers used in RT-PCR.

Target genes	Primers
GAPDH	5’ AGTGCCAGCCTCGTCTCATAG 3’
	5’ CGTTGAACTTGCCGTGGGTAG 3’
HIF-1α	5’ CTCCCATACAAGGCAGCAGAAA 3’
	5’ CAAAACAACCAACAGAAACGAAAC 3’
TGF-β1	5’ GTGGCTGAACCAAGGAGACG 3’
	5’ CAGGTGTTGAGCCCTTTCCAG 3’

### Statistical Analysis

The results were analyzed using the SPSS 17.0 statistical software. Data were expressed as the means ± SE (standard error) for each group. The one-way analysis of variance (ANOVA) was used for comparisons between groups, and the LSD method was used for pairwise comparisons. P value<0.05 (2-sided) was considered to be statistically significant.

## Results

No rat died during the study. A few rats experienced peritoneal sclerosis and injection difficult at the late stage, and the IP injection treatment plan was completed successfully after changing injection sites. No significant difference was found in body weight of rats in each group (P>0.05) at the beginning of the experiment (d0). And the weights of rats increased gradually with time. At d7 (the seventh days of IP injection), d14, d21 and d28, there were no significant difference in body weights of rats between each two groups (P>0.05).

### Impact of LMWH on UF and peritoneal transportation function (Table 2)

UF, and D2/D0 ratio of group GLU were significantly lower than that of group CON and group NS (p<0.05). However, UF and D2/D0 of groups HEP, LLMWH and HLMWH were higher than that of group GLU (p<0.05). And UF of group HLMWH was higher than that of group LLMWH (Table 2). The D/Purea (dialysate-to-plasma ratios of urea), D/Palb (dialysate-to-plasma ratios of albumin), D/Ptb (dialysate-to-plasma ratios of total protein) and WBC (numbers of white blood cells in peritoneal dialysate) of group GLU was significantly higher than that of group CON and group NS (p<0.05). However, the D/Purea, D/Palb, D/Ptb and WBC of groups HEP, LLMWH and HLMWH were lower than that of group GLU (p<0.05). No difference in D/Purea, D/Palb, D/Ptb and WBC was found between group LLMWH and group HLMWH (Table 2).

**Table 2:** Peritoneal Function of each group (X̅±s).

Group	Case	UF(ml)	D2/D0	D/Purea	D/Palb×1000	WBC(10^6^/L)*
CON	6	12.60±1.04	0.92±0.05	0.52±0.05	20.65±4.97	168±48
NS	10	12.23±1.13	0.83±0.05^▲^	0.55±0.08	30.40±7.99^▲^	286±112^▲^
GLU	10	6.55±1.08^▲^ ^■^	0.55±0.11^▲^ ^■^	0.75±0.05^▲^ ^■^	65.66±9.53^▲^ ^■^	738±167^▲^ ^■^
HEP	6	7.47±1.05^★^	0.77±0.05^▲^ ^★^	0.64±0.06^▲^ ^■^ ^★^	57.65±5.32^▲^ ^■^ ^★^	547±138^▲^ ^■^ ^★^
LLMWH	10	7.63±0.95^★^	0.76±0.12^▲^ ^★^	0.66±0.06^▲^ ^■^ ^★^	55.66±7.54^▲^ ^■^ ^★^	601±239^▲^ ^■^
HLMWH	10	9.70±0.96^★^ ^●^	0.80±0.07^▲^ ^★^	0.64±0.06^▲^ ^■^ ^★^	54.58±5.67^▲^ ^■^ ^★^	476±203^▲^ ^■^ ^★^

*WBC: Numbers of white blood cells in peritoneal dialysate; UF: ultrafiltration volume;

^▲^P<0.05 vs. group CON;

^■^P<0.05 vs. group NS;

^★^P<0.05 vs. group GLU;

^●^P<0.05 vs. group LLMWH.

### Impact of LMWH on blood coagulation function

After IP injection of heparin and LMWH for 28 days, no significant change in Prothrombin Time (PT) and Activated Partial Thromboplastin Time (APTT) was revealed (P>0.05) in each group.

### Impact of LMWH on peritoneal morphological appearance

**Fig 1:**
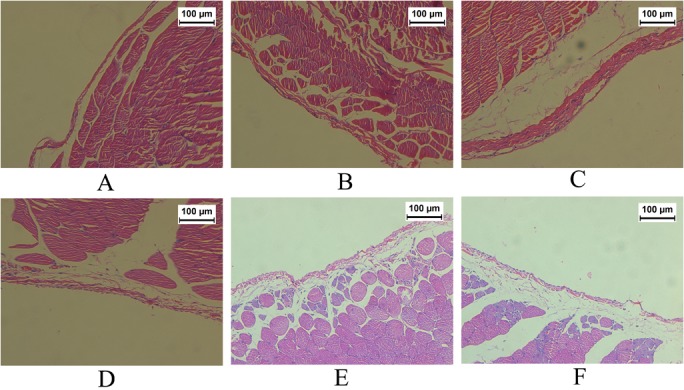
Hematoxylin and Eosin (HE) staining of the parietal peritoneum (×200). Group CON (A), group NS (B), group GLU (C), group HEP (D), group LLMWH (E) and group HLMWH (F).

In HE staining, parietal peritoneum of group CON was covered with a layer of mesothelial cells. The collagen matrix under the mesothelial cells was thin. Fibroblasts, inflammatory cells and blood vessels were rare in the submesothelial compact zone. The peritoneal morphological appearance of group NS was similar to that of group CON. Group GLU was associated with severe loss of mesothelial cells, increased thickness of the submesothelial compact zone, and marked proliferation of fibroblasts, inflammatory cells and neovessels. However, such pathological changes ameliorated significantly in groups HEP, LLMWH and HLMWH (Fig. 1).

**Fig 2:**
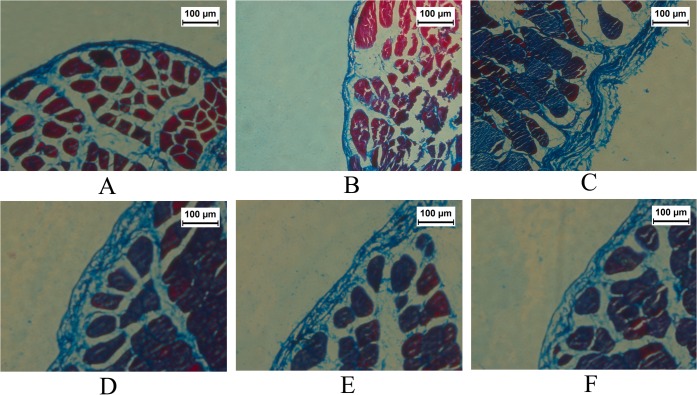
Masson Trichrome Staining of the parietal peritoneum (×200). Group CON (A), group NS (B), group GLU (C), group HEP (D), group LLMWH (E) and group HLMWH (F).

**Fig 3:**
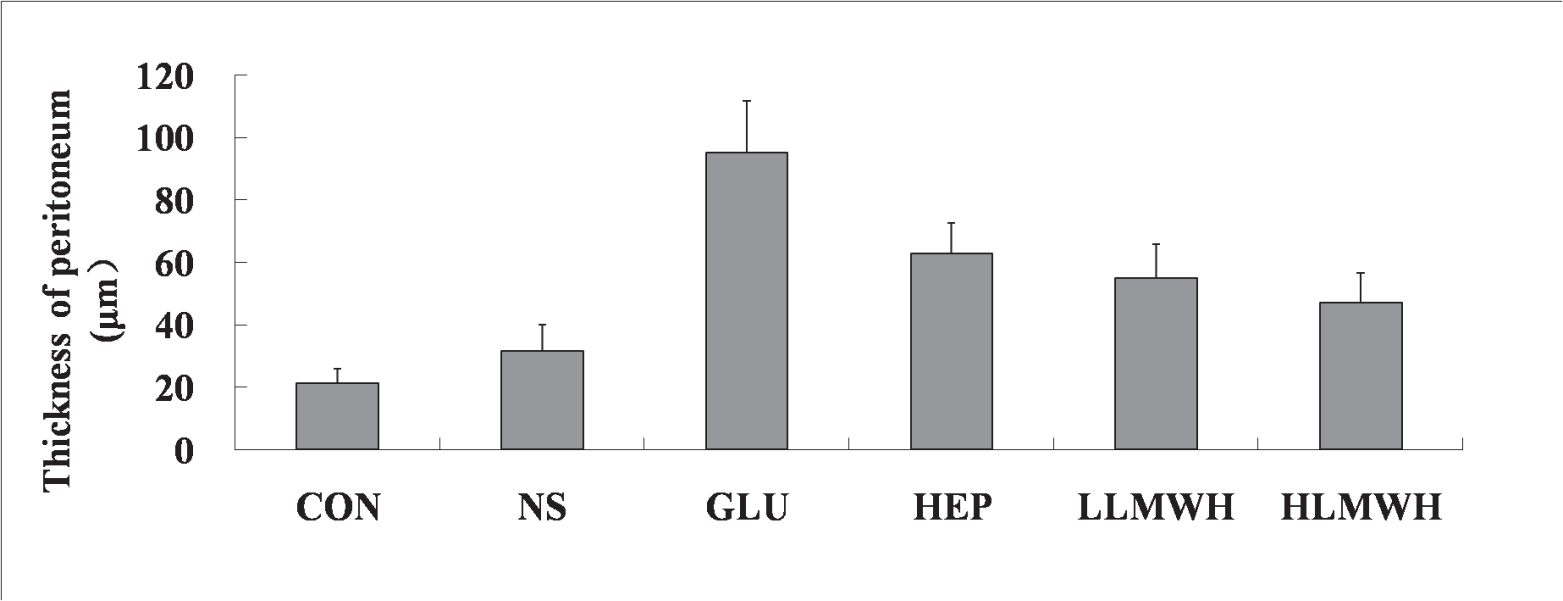
Thickness of parietal peritoneum evaluated under Masson Trichrome Staining. ▲P<0.05 vs. group CON; ■P<0.05 vs. group NS; ★P<0.05 vs. group GLU; ◆P<0.05 vs. group HEP;●P<0.05 vs. group LLMWH.

In Masson Staining, the peritoneal thickness of group GLU was significantly higher than that of group CON and group NS (p<0.05). However, compared with group GLU, it decreased significantly in groups HEP, LLMWH and HLMWH (p <0.05). The peritoneum of group HLMWH was thinner than that of group HEP. No difference in peritoneal thickness was found between group LLMWH and group HLMWH (Fig. 2 and Fig. 3).

### Impact of LMWH on neoangiogenesis in mesentery and greater omentum

**Fig 4:**
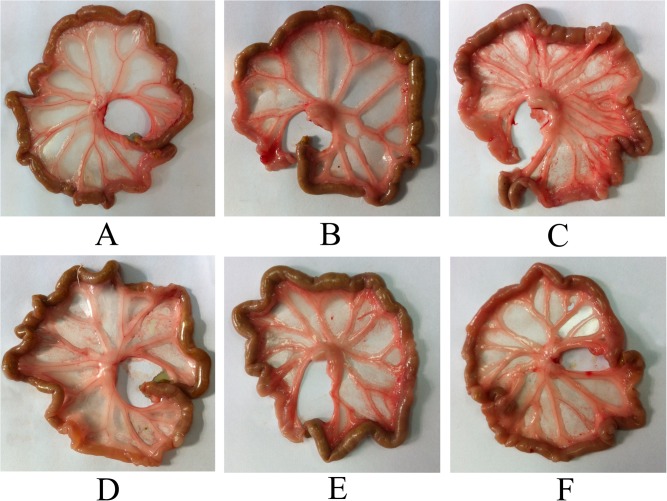
Gross appearance of mesentery under direct view. A: Group CON, transparent mesentery with few small vessels; B: group NS, transparent, with slight hyperplasia of mesenteric vessels; C: group GLU, translucency, with obvious hyperplasia of mesenteric vessels and white fibrous material deposition at the origin of mesenteric artery; D: group HEP, translucency, with obvious hyperplasia of mesenteric vessels; E: group LLMWH, transparent, with slight hyperplasia of mesenteric vessels; F: group HLMWH, transparent, with slight hyperplasia of mesenteric vessels.

**Fig 5:**
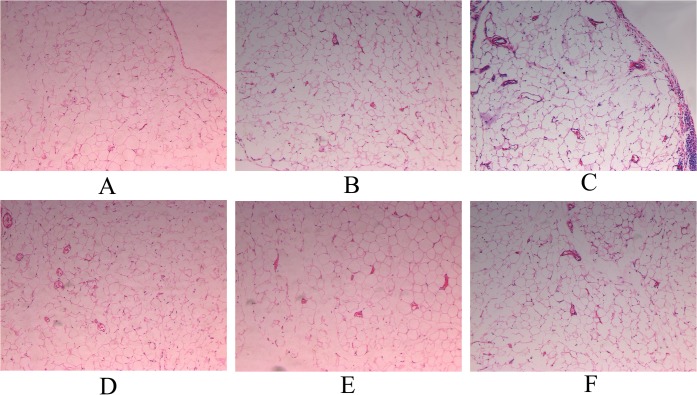
Numbers of neovessels in greater omentum under microscope (HE staining, ×100). Group CON (A), group NS (B), group GLU (C), group HEP (D), group LLMWH (E) and group HLMWH (F).

To reveal the impact of LMWH on neoangiogenesis in peritoneum, the gross appearance of mesentery were observed under direct view and the numbers of neovessels in greater omentum were studied under microscope. Our experiments revealed that group GLU was associated with obvious hyperplasia of mesenteric vessels and white fibrous material deposition at the origin of mesenteric artery, the IP administration of heparin and LMWH significantly alleviated these alterations (Fig. 4, P<0.05). The numbers of neovessels in greater omentum of group GLU (8.54±2.06/mm^2^) was significantly higher than that of group CON (1.42±0.34/mm^2^)and group NS (2.50±0.84/mm^2^), and it decreased significantly in group HEP (4.97±0.48/mm^2^), group LLMWH (4.33±0.66/mm^2^) and group HLMWH (3.40±0.67/mm^2^) (Fig. 5, p<0.05). The numbers of neovessels in greater omentum of group HEP was significantly higher than that of group HLMWH (p<0.05).

### Expression of HIF-1α, VEGF and TGF-β1 in the parietal peritoneum by IHC

**Fig 6:**
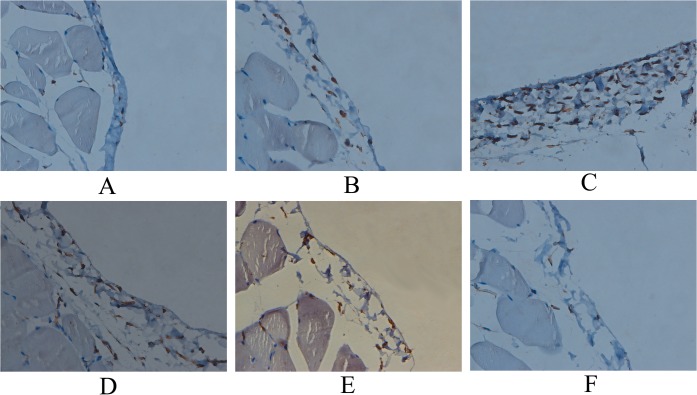
Expression of HIF-1α in parietal peritoneum (×400). Group CON (A), group NS (B), group GLU (C), group HEP (D), group LLMWH (E) and group HLMWH (F).

Positive cells of HIF-1α, VEGF and TGF-β1 were occasionally found in the peritoneum of group CON and group NS. The number of HIF-1α, VEGF and TGF-β1 positive cells increased significantly in group GLU (all p<0.05). The numbers of HIF-1α, VEGF and TGF-β1 positive cells in groups HEP, LLMWH and HLMWH reduced significantly (all p<0.05), and the decreases in groups LLMWH and HLMWH were much greater than that in group HEP (all p<0.05). The numbers of VEGF and TGF-β1 positive cells in group HLMWH were much lower than that in group LLMWH (Fig. 6, Table 3, all p<0.05).

**Table 3:** The staining score of HIF-1α, VEGF and TGF-β1 in peritoneum by IHC (X̅±s).

Group	Case	HIF-1α	VEGF	TGF-β1
CON	6	0.19±0.014	0.26±0.08	0.21±0.03
NS	10	0.39±0.08	0.35±0.08	0.40±0.08^▲^
GLU	10	2.54±0.33^▲^ ^■^	2.41±0.52^▲^ ^■^	2.34±0.38^▲^ ^■^
HEP	6	1.67±0.32^▲^ ^■^ ^★^	1.77±0.37^▲^ ^■^ ^★^	1.76±0.41^▲^ ^■^ ^★^
LLMWH	10	1.04±0.33^▲^ ^■^ ^★^ ^◆^	1.45±0.28^▲^ ^■^ ^★^ ^◆^	1.36±0.43^▲^ ^■^ ^★^ ^◆^
HLMWH	10	0.89±0.22^▲^ ^■^ ^★^ ^◆^	0.91±0.23^▲^ ^■^ ^★^ ^◆^ ^●^	0.92±0.25^▲^ ^■^ ^★^ ^◆^ ^●^

^▲^P<0.05 vs. group CON;

^■^P<0.05 vs. group NS;

^★^P<0.05 vs. group GLU;

^◆^P<0.05 vs. group HEP;

^●^P<0.05 vs. group LLMWH.

### Expression levels of HIF-1α and TGF-β1 mRNA in the peritoneum by RT-PCR

**Fig 7:**
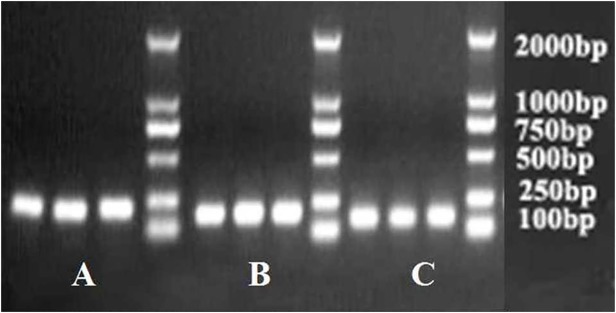
The electropherogram of RT-PCR product of GAPDH(A), TGF-β1(B), and HIF-1α (C).

The mRNA expression levels of HIF-1α and TGF-β1 in the parietal peritoneum were evaluated by RT-PCR. The electropherogram of RT-PCR product of HIF-1α and TGF-β1 were showed in Fig. 7. It was demonstrated that the mRNA expression levels of HIF-1α and TGF-β1 in group GLU, were much higher that in group CON and group NS (Table 4, all p<0.05). And the mRNA level of HIF-1α and TGF-β1 decreased significantly in groups HEP, LLMWH and HLMWH (Table 4, all p<0.05). And the mRNA level of HIF-1α and TGF-β1 in group HLMWH is much lower than that in group HEP, and the mRNA level of HIF-1α and TGF-β1 in group HLMWH is much lower than that in group LLMWH (Table 4, all p<0.05).

**Table 4:** Relative expression level of HIF-1α and TGF-β1 mRNA in peritoneum(X̅±s).

Group	Case	HIF-1α	TGF-β1
CON	6	1.00±0.00	1.00±0.00
NS	10	2.65±0.78^▲^	2.57±0.61^▲^
GLU	10	9.91±2.23^▲^ ^■^	6.46±1.60^▲^ ^■^
HEP	6	5.78±1.03^▲^ ^■^ ^★^	4.94±1.39^▲^ ^■^ ^★^
LLMWH	10	4.86±0.58^▲^ ^■^ ^★^	4.32±0.49^▲^ ^■^ ^★^
HLMWH	10	2.70±0.54^▲^ ^★^ ^◆^ ^●^	2.93±0.45^▲^ ^★^ ^◆^ ^●^

^▲^P<0.05 vs. group CON;

^■^P<0.05 vs. group NS;

^★^P<0.05 vs. group GLU;

^◆^P<0.05 vs. group HEP;

^●^P<0.05 vs. group LLMWH.

## Discussion

LMWH belongs to the family of glycosaminoglycans (GAGs). It inhibits the coagulation process by binding to antithrombin, and then accelerates antithrombin’s inhibition of activated factor X. IP injection of LMWH is commonly used in a setting of peritonitis in end-stage renal disease patients undergoing CAPD, mostly to diminish fibrin deposition and to prevent the occlusion of the peritoneal catheter and intra-abdominal adhesions[10]. Besides its anticoagulant activities, it also had pleiotropic effects, including modulation of cell growth and proliferation via activity on numerous growth factors affecting inflammation, fibrosis and vasculopathy [10,11]. A growing body of evidence showed that IP injection of LMWH to CAPD patients could ameliorate peritoneal membrane structure and peritoneal function[10,12]. Sulodexide (a type of LMWH) was found to improve peritoneal function by increasing the D/Purea and by decreasing peritoneal protein loss[13], and to improve histological alterations of the peritoneal membrane in rat peritoneal sclerosis model[14]. In a word, IP injection of LMWH may improve, prevent, or even treat peritoneal membrane damage in long-term CAPD patients. It was reported that LMWH could preserve peritoneal membrane since it may inhibit the process of inflammation, neoangiogenesis and fibrosis, by blunting the activity of glucose degradation products (GDPs), suppressing the expression of VEGF and TGF-β1, and upregulation of HGF (hepatocyte growth factor) [10]. However, the exact mechanism of its influence on peritoneal structure and function in CAPD patients is still not completely clear.

In our study, the rat peritoneal fibrosis model was established by daily IP administration of PDF and Erythromycin Lactobionate to observe the effect of LMWH on peritoneal function and peritoneal fibrosis. Our study revealed that, compared with group CON and group NS, the UF and D2/D0 decreased significantly, and the D/Purea, D/Palb and numbers of WBC in peritoneal dialysate (shorted as WBC) increased significantly in group GLU. After IP administration of heparin or LMWH, the above-mentioned alterations ameliorated significantly. The D2/D0, D/Purea, D/Palb and WBC of group LLMWH and group HLMWH did not exhibit any significant difference. However, the UF in group HLMWH was significantly higher than that in group LLMWH, indicating that the increased dose of LMWH would result in further improvement of UF. Our results were consistent with the study of Schilte [1] and Sjoland [7]. Schilte [1] found that rats received 5 weeks treatment of conventional PDF were associated with reduced UF, increased glucose absorption and elevated permeability. But these adverse changes were not ameliorated after the administration of heparin or LMWH [1]. Sjoland[7] showed that UF and D4/D0 (4 hours-to-0 hour ratio of glucose in dialysate) increased significantly after IP administration of LMWH. Taken together, our study showed that peritoneal permeability to glucose, urea, albumin and WBC increased markedly after long term exposure to PDF with high glucose, resulting in reduced UF and damaged peritoneal transport function. The IP injection of heparin and LMWH could ameliorate these adverse alterations.

In order to explore the role of LMWH on peritoneal structure, the morphological appearance and neoangiogenesis of mesentery, omentum and peritoneum were studied. Local sterilized inflammation may initiate the process of neoangiogenesis and fibrosis, and eventually lead to ultrafiltration failure. Similar to previous reports[2], our study indicated that the peritoneum in group GLU was associated with loss of mesothelial cells, proliferation of fibroblasts, inflammatory cells and neovessels. All of these phenomena were regarded as indicators of inflammation. That is to say, group GLU was associated with increased inflammation in peritoneum. Peritoneal thickness, being a feature of fibrosis, is the results of fibroblast proliferation and extracellular matrix(ECM) deposition. Our results found that the thickness of peritoneum increased significantly in group GLU in HE and Masson staining. Neoangiogenesis is also an important factor predisposing to increased peritoneal permeability and ultrafiltration failure. Neovessels increased the exchange area of the vascular bed and the transport rate of small molecules, which may accelerate the absorption of small molecular solutes, such as urea and glucose. The increase in peritoneal permeability and transport function eventually led to ultrafiltration failure [2]. To reveal the impact of LMWH on neoangiogenesis in peritoneum, the mesentery was observed under direct view, and group GLU was found to be associated with obvious hyperplasia of mesenteric vessels, Furthermore, numbers of neovessels in omentum were studied under microscope, with group GLU having more neovessels than that of group CON and group NS. More importantly, all these above mentioned changes ameliorated significantly in groups LLMWH and HLMWH. In brief, our findings indicated that IP administration of LMWH could improve peritoneal function by inhibiting inflammation, fibrosis and neoangiogenesis in peritoneum.

Both of heparin and LMWH were all reported to ameliorate peritoneal function and structure. But there is no conclusion on the issue “whose effect is better”. Our study demonstrated that, no significant difference was found in UF, D2/D0, D/Purea, D/Palb and WBC between group HEP and group LLMWH (or group HLMWH). However, group HLMWH was significantly lower than group HEP in peritoneum thickness and neoangiogenesis in mesentery and greater omentum. That is to say, LMWH had the same effect as heparin on peritoneal function, but it is superior to heparin in ameliorating peritoneal structure. In addition, heparin has potential harm on platelet function and blood clotting, although it hadn’t been proved by our study. Furthermore, LMWH is also superior to heparin in terms of improved bioavailability, prolonged half-life, improved efficacy and minimal interference with normal blood coagulation function. Taken together, LMWH is superior to heparin when they were IP administrated to improve peritoneal function and inhibit peritoneal fibrosis.

The exact molecular mechanism of LMWH’s influence on peritoneal structure and function is still unknown. Our study showed that the numbers of HIF-1α, VEGF and TGF-β1 positive cells increased significantly in group GLU. The reduction in HIF-1α, VEGF and TGF-β1 positive cells in groups HEP, LLMWH and HLMWH suggested that heparin and LMWH suppressed the expression levels of the three genes. The VEGF and TGF-β1 levels in group HLMWH were significantly lower than that in group LLMWH (P<0.05), which indicated that LMWH might inhibit the expression of VEGF and TGF-β1 in a dose-dependent manner. The HIF-1α level in group HLMWH was slightly lower than that in group LLMWH without statistical significance (P>0.05), which may have something to do with the relative small sample size. RT-PCR also proved that LMWH can significantly decrease HIF-1α and TGF-β1 in a dose-dependent way, since the mRNA levels of HIF-1α and TGF-β1 in group HLMWH were significantly lower than that in group LLMWH (P<0.05). Our study also revealed that group HLMWH was significantly lower than group HEP in the levels of HIF-1α, VEGF and TGF-β1 detected by IHC and RT-PCR. Taken this fact that “LMWH is a subunit of heparin” into consideration, our results suggested that both LMWH and heparin might take effect in the same mechanism, and that it might be LMWH that had direct effect on peritoneal function and fibrosis.

LMWH was found to alleviate inflammation and fibrosis in rats’ lung tissue through inhibiting the expression of HIF-1α and VEGF [9]. Chronic hypoxia is an important reason to promote peritoneal fibrosis and ultrafiltration failure [5]. HIF-1α, a inducible transcription factor in hypoxia condition, plays an important role in angiogenesis, inflammatory reaction, ECM deposition and fibrosis of peritoneum [6]. Studies had shown that cells deficient in HIF-1α were not able to produce Connective Tissue Growth Factor (CTGF) mRNA, which played a major role in pathways that lead to fibrosis, including fibrosis of major organs and fibroproliferative diseases [15]. VEGF, which is also the downstream target genes of HIF-1α, is a well-known factor stimulating neoangiogenesis [16]. TGF-β1 is closely related with extracellular matrix (ECM) deposition and tissue fibrosis, and it is recognized as a well-known index reflecting the extent of peritoneal fibrosis [17,18]. Firstly, TGF-β1 can regulate the proliferation, differentiation and apoptosis of cell, and accelerate the synthesis of ECM; Secondly, TGF-β1 could stimulate the formation of ECM components, such as collagen and proteoglycan directly, and inhibit the degradation of ECM; Furthermore, TGF-β1 can promote the adhesion and deposition ability of matrix [19]. TGF-β1 was a potent fibrosis factor through the activation of interleukin-6 (IL-6) in fibroblast. Ectogenic overexpression of TGF-β1 resulted in a series of changes, such as peritoneal thickening, reduced UF, ECM deposition, VEGF expression neoangiogenesis, and peritoneal fibrosis [2]. TGF-β1 plays an important role in the accumulation of peritoneal extracellular matrix and peritoneal fibrosis [20]. The TGF-β1/Smad pathway in peritoneal fibrosis is stimulated by PD solutions [21]. TGF-β1 production can also stimulate cardiac fibrosis [22]. The acting mechanisms between HIF-1α and TGF-β1 are complex. Shih[23] identified that TGF-β1 stimulated the expression of HIF-1α and its downstream gene VEGF in fibrosarcomas. Qian[24] reported, TGF-β1 induced HIF-1α mediated VEGF secretion in normal human cytotrophoblast cells. However, HIF-1α could also promote angiogenesis and fibrosis of peritoneum in a TGF-β1 independent mechanism [25]. The effects of TGF-β1 in pulmonary fibrosis is dependent on HIF-1α. HIF-1α silencing attenuated the expression of TGF-β1 induced pro-fibrotic genes, including platelet-derived growth factor-A (PDGF-A) plasminogen activator inhibitor-1 (PAI-1) [26]. Subeq [27] reported that intravenous administration of 3 mg/kg/day valsartan ameliorated chlorhexidine digluconate-induced peritoneal fibrosis by decreasing serum and dialysate TGF-β1 levels and significantly decreased the expression of TGF-β1, α-SMA, fibronectin, collagen and VEGF in rats’ peritoneum.

In conclusion, our study demonstrated that IP administration of LMWH could improve peritoneal function and relieve peritoneal fibrosis, possibly through suppression of HIF-1α, VEGF and TGF-β1. These findings provide novel research direction and therapeutic targets for peritoneal fibrosis. Further experiments are needed to determine whether LMWH directly inhibit the expression of HIF-1α, VEGF and TGF-β1, and to explore the exact molecular mechanism among the three genes.
